# Endocarditis due to *Aggregatibacter Segnis*: a rare case report

**DOI:** 10.1186/s12879-023-08231-x

**Published:** 2023-05-08

**Authors:** Xiaoxiao Guo, Xinyun Zhang, Yanli Qin, Hong Liu, Xinyu Wang

**Affiliations:** 1 Department of Infectious Diseases, Shanghai Key Laboratory of Infectious Diseases and Biosafety Emergency Response, Shanghai Medical College, National Medical Center for Infectious Diseases, Huashan Hospital, Fudan University, Shanghai, China; 2 Department of Laboratory Medicine, Shanghai Medical College, Huashan Hospital, Fudan University, Shanghai, China

**Keywords:** *Aggregatibacter segnis*, HACEK, Infective endocarditis

## Abstract

**Background:**

As a member of the HACEK group, *Aggregatibacter segnis* (*A. segnis*) is a fastidious Gram-negative coccobacillus that resides in the human oropharyngeal flora. Infective endocarditis caused by *A. segnis* is rarely reported.

**Case presentation:**

A 31-year-old male was admitted to our hospital for a 3-month history of intermittent high fever, chills, and chest distress. On presentation, he was febrile and tachycardic but otherwise with stable vital signs. Physical examination revealed systolic murmurs in the aortic and mitral valve areas. Pitting edema was evident in the lower extremities. Transthoracic echocardiography demonstrated multiple vegetations in the mitral and aortic valves. Severe regurgitation of the aortic valve and left heart dysfunction were also detected. With the suspicion of infective endocarditis and heart failure, we immediately performed microbiological tests and arranged the cardiac replacement surgery. Matrix-assisted laser desorption ionization-time of flight (MALDI-TOF) mass spectrometry and metagenomic next-generation sequencing (mNGS) identified *A. segnis* from the bloodstream. While the surgical specimen culture was negative, the mNGS was positive for *A. segnis*. The patient was treated with ceftriaxone for four weeks and discharged. He remained clinically well, with laboratory results restored.

**Conclusion:**

This is the first report of *A. segnis* infective endocarditis that combined MALDI-TOF and metagenomic next-generation sequencing in the diagnosis. The hypothesis-independent molecular techniques can outperform conventional tools to prevent diagnostic delay.

## Background

Infective endocarditis (IE) is anx` uncommon disease with diverse presentations and a poor prognosis [1]. The acronym HACEK denotes a group of Gram-negative fastidious organisms, including *Haemophilus* and *Aggregatibacter* spp., *Cardiobacterium* spp., *Eikenella corrodens*, and *Kingella* spp. [2, 3], which are commensals in the oropharyngeal, respiratory, gastrointestinal, and urogenital tract. The HACEK group contributes to most Gram-negative bacterial endocarditis, accounting for 1.2-3% of all IE cases [4, 5]. However, the fastidious feature often challenges traditional microbiological procedures, which may result in diagnostic delay and disease deterioration.

*Aggregatibacter segnis*, formerly known as *Haemophilus segnis*, was first isolated from human dental plaque in 1976. The genus *Aggregatibacter* was created in 2006 to accommodate some species that were distantly related to the *Haemophilus* genus [6, 7]. *A. segnis* is an uncommon commensal in the oropharynx, and rarely has it been reported as the cause of endocarditis. In this case report, we present a rare case of endocarditis infected by *A. segnis* and the first successful bacteria identification from both peripheral blood and valve vegetations.

## Case presentation

A 31-year-old male was admitted to the Infectious Disease department on May 19, 2021, for a 3-month history of intermittent high fever up to 40 °C, chills, and occasional dyspnea. Three months ago, he developed fever with chills, and went to a local emergency department. The blood test showed a significant increase in white blood cell counts (23.0 × 10^9^/L, neutrophils: 94%) and C-reactive protein (CRP, 54 mg/L). He was prescribed cephalosporin, which helped subside the fever. However, the fever recurred 20 days later. Reexamination of laboratory tests revealed a white blood cell count of 13 × 10^9^/L with 79% neutrophils. The concentration of procalcitonin (PCT) and CRP was 1.23 ng/mL and 120.0 mg/L, respectively. The erythrocyte sedimentation rate was 65 mm/h. The patient’s body temperature went normal after the treatment of levofloxacin and latamoxef. One month later, he had fever again with progressive shortness of breath. The patient went to our hospital and was hospitalized for further investigation. No history regarding pets, travel, drug abuse, dental diseases or procedures was reported. On the physical examination, the pulse was regular at 108 beats per minute, and the blood pressure was normal. Grade 3/6 systolic murmurs were audible in the aortic and mitral valve areas. No petechiae were noted in his conjunctiva and oral mucosa. Roth spots were not detected. Pitting edema was evident in the lower extremities. No neurologic deficits or skin rashes were observed.

During hospitalization, blood tests demonstrated as follows: white blood cell count, 14.5 × 10^9^/L (neutrophils: 85.4%, lymphocytes: 10%); hemoglobin, 131 g/L; platelet count, 409 × 10^9^/L; CRP, 80.1 mg/L; and PCT, 1.23ng/ml. Other laboratory tests were notable for a troponin T level of 0.748 ng per milliliter (reference range, < 0.1) and an N-terminal B-type natriuretic peptide (NT-BNP) level of 6230 pg per milliliter (reference range, < 100). The urinalysis reported: erythrocyte (1.3/µl), leukocytes (2.6/µl), occult blood (-). The rheumatoid factor (RF) tests showed elevated levels of RFIgA (131.7 U/ml) and RFIgM (31.3 U/ml). The renal and liver function tests were within the normal ranges.

**Fig. 1:**
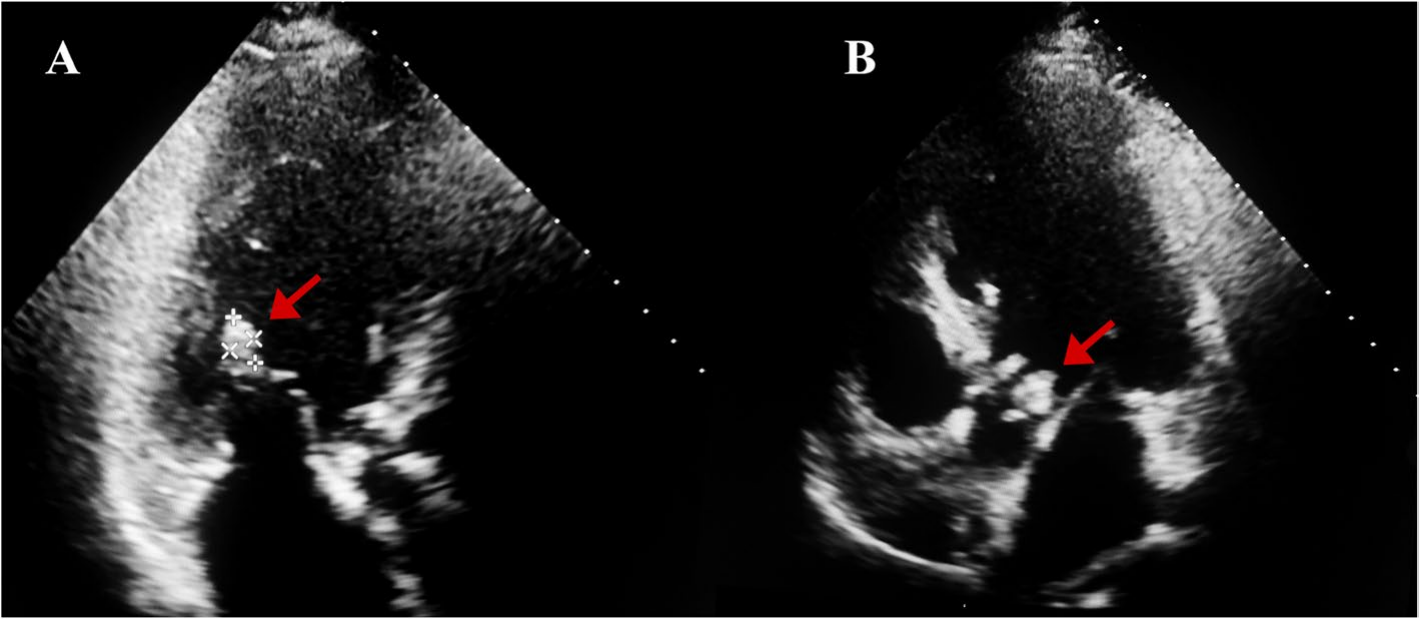
Transthoracic echocardiogram showing multiple vegetations. **A** The representative vegetations (arrow) in the mitral valve. **B** A 9 mm × 6 mm vegetation (arrow) in the aortic valve

The electrocardiogram showed sinus tachycardia and ST-T changes (1-2.5 mm ST-segment depressions in leads I, aVL, V3, V4, V5, and V6; a subtle T wave inversion in lead V3; T wave inversions in leads I, aVL, V4, V5, and V6). Chest computerized tomography (CT) scan suggested lung inflammation, pleural effusion, and lymphadenopathy in the mediastinum. The abdominal CT did not find obvious abnormalities in the liver, kidneys, or spleen. Transthoracic echocardiography was performed. In the mitral valve, the chordae tendineae were elongated and seeded by multiple vegetations, of which the largest was 9 mm × 6 mm. A 23 mm × 10 mm semilunar anechoic zone in the aortic root was found. Striped vegetations, abscesses, and severe regurgitation were detected in the aortic valve (Fig. 1). The left ventricular ejection fraction was 48%.

**Fig. 2:**
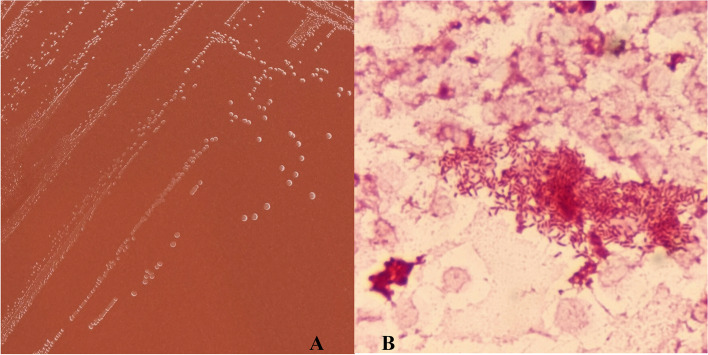
The microbiological findings in the blood agar culture and Gram staining. **A** Colonies of the isolate on the chocolate agar. **B** Microscopic examination of a Gram-stained smear reveals small Gram-negative coccobacilli

Two sets of blood cultures were obtained before antibiotic therapy. Meanwhile, considering the patient’s symptoms of cardiac insufficiency and multiple valve vegetations, we contacted the surgery department immediately to arrange a valve surgery. Both aerobic bottles confirmed positivity within 24 h. A subculture was performed on chocolate agar (Comagal, Shanghai, China) at 35 °C with 5% CO_2_. The isolates of coccobacilli (Fig. 2) were identified as *Aggregatibacter spp.* The strain was further analyzed by matrix-assisted laser desorption ionization-time of flight mass spectrometry (MALDI-TOF, Vitek MS, V3.2 Database, bioMérieux, France), which provided a 99.9% matching similarity against *A. segnis*. The isolates were sensitive to amoxicillin, cefuroxime, ceftizoxime, levofloxacin, azithromycin, and sulfamethoxazole. Metagenomic next-generation sequencing (mNGS) of peripheral blood also identified *A. segnis* (731 sequences).

Based on Duke Criteria, the patient’s final diagnosis was confirmed to be infective endocarditis caused by *A. segnis*. Ceftriaxone was prescribed for antimicrobial treatment. Metoprolol was used to control the tachycardia and palpation. Diuretics were administered to decrease cardiovascular congestion. When the condition stabilized, the patient received aortic valve replacement surgery. Part of the vegetation was collected for conventional culture and mNGS. While the surgical specimen culture was negative, the mNGS was positive for *A. segnis* (929 sequences).

After the operation, the patient was treated with ceftriaxone for another four weeks and discharged. He remained clinically well in the outpatient follow-up, with laboratory results restored .

## Discussion and conclusion

The HACEK group bacteria, fastidious Gram-negative organisms, account for 1.2-3% of all infective endocarditis cases. Compared to other causative pathogens, HACEK group endocarditis tends to be characterized by an insidious course with a mean duration of about 13 weeks of symptoms before diagnosis [4]. Diagnosis of infective endocarditis is challenging due to non-specific clinical manifestations and effective short-term empirical antibiotic treatment. Positive findings in physical examination warrant further investigation of suspected infective endocarditis: audible murmurs, petechiae and cutaneous infarcts, splenomegaly, neurovascular changes, Roth spots, etc [8]. Once infective endocarditis is suspected, echocardiography, especially transesophageal echocardiography, is recommended to locate vegetations, evaluate cardiac functions, and assess surgical indications.

In this case, the patient experienced recurrent episodes of hyperthermia, which indicated the presence of an infection, as confirmed by elevated levels of inflammatory biomarkers. The hypothesis of bacterial infections was supported by the effectiveness of short-term antibiotic treatment in relieving symptoms and restoring white blood cell count. Unfortunately, the final diagnosis was delayed until three months later, when echocardiogram and pathogen identification confirmed the condition.

*A. segnis*, a gram-negative coccobacillus, is recognized as a commensal in the oropharynx and is rarely encountered in infective endocarditis. This case report presents the first direct identification of *A. segnis* from cardiac valve vegetations, which has not been previously documented. In 1988, Bansborg et al. published the first case of *Haemophilus* (now *Aggregatibacter*) *segnis* endocarditis [9]. The patient was a hitherto healthy 76-year-old female with a three-month history of weight loss and cutaneous abscesses on the extremities. An extended period of blood culture and cross immunoelectrophoresis helped define the causative organism. The second report of *A. segnis* endocarditis was published in 2003, where a 59-year-old male was admitted to the hospital for a six-week history of intermittent headache and slurred speech. Transesophageal echocardiography suggested endocarditis, while API bacterial identification and 16 S rRNA sequencing confirmed the A. segnis isolate from blood samples [10].

Both the two cases and ours experienced a medical history of more than one month of subacute symptoms. On arrival, all three patients complained about different discomforts. The first case and ours were febrile and malaise, whereas the second case mainly suffered from headache and slurred speech. Fever is reported in 80% of infective endocarditis cases, with a higher occurrence rate in those caused by the HACEK group [11, 12]. The three patients shared common examination findings including murmurs and echocardiographic evidence supporting endocarditis, which are essential indicators for subacute infective endocarditis [13, 14]. Only our case underwent heart valve replacement surgery, and all cases had a favorable overall outcome.

The prevalence of the HACEK group may have been underestimated due to their slow growth rate and the phenotypic resemblance to other strains. However, there have been tremendous advancements in organism identification over the past few decades. MALDI-TOF mass spectrometry has recently offered a rapid and accurate method to identify microbes within minutes, including HACEK strains [15–17]. Compared to the conventional phenotypic means, MALDI-TOF mass spectrometry performs better in identifying slowly growing fastidious bacteria like *A. actinomycetemcomitans* [18]. Despite the different protein spectra between *A. segnis* and the other two *Aggregatibacter* species, the application of MALDI-TOF is worthy of further exploration. Conventional tests to discriminate *A. segnis* from species of *Haemophilus* and *Aggregatibacter* is time-consuming and laborious. Our case firstly reported the delineation of *A. segnis* by MALDI-TOF mass spectrometry from an isolate of blood culture. MALDI-TOF mass spectrometry could be a promising alternative method for routine identification of the HACEK group.

Prior antibiotic exposure and the subacute course of infective endocarditis further contribute to culture-negative endocarditis. Culture-independent DNA-based methods were recommended in the etiological diagnosis of infective endocarditis [19]. 16 S rRNA gene sequencing used to identify and classify fastidious organisms accurately. The 16 S rRNA gene sequencing application in defining *A. segnis* bacteremia showed higher efficiency and accuracy than conventional phenotypic tests or the Vitek System [20]. However, 16 S rRNA gene sequencing is applied to detect pathogens with matched primers, which may reduce the specificity in diagnosing infections caused by rare bacteria. mNGS is a high-throughput approach that provides a comprehensive analysis of the microbial community in clinical samples [21]. Studies have shown that mNGS can detect pathogens in the bloodstream when the patients are on antimicrobial therapy, raising the possibility of etiological diagnosis in culture-negative cases [22, 23]. Vegetations are loaded with significantly higher-grade bacteria than blood culture; as a result, the culture of valve tissue can assist in microbiologic diagnosis [11]. Compared to blood cultures, mNGS detection of valve tissue also provides a high sensitivity between 85.7% and 100%, and the specificity is 72.7-100% [24–26]. In a previous case report [20], *A. segnis*-related endocarditis was identified by phenotypic testing and 16 S rRNA polymerase chain reaction (PCR) in the blood sample. In our case, blood cultures were applied before empiric antibiotic treatment. We first diagnosed *A. segnis*-related endocarditis by MALDI-TOF mass spectrometry in blood culture. Then, we used next-generation sequencing analysis in the peripheral blood and vegetation to confirm the diagnosis, which was never reported. The combination of MALDI-TOF and mNGS can effectively complement and reinforce each other, resulting in the identification of fastidious bacteria with high accuracy within a short timeframe. Additionally, the increasing adoption and promotion of mNGS has made this approach more cost-effective.

We report a rare case of *A. segnis* infective endocarditis detected by a circumspect examination and the assistance of hypothesis-free pathogenic detection tools. A definite diagnosis of IE caused by HACEK bacteria may be hard to establish, in which case the fastidious pathogens are difficult to isolate in culture. MALDI-TOF and mNGS can provide alternative tools to identify microbiological evidence in a short turnaround time to prevent further pathogenic invasion.

## Data Availability

All the data that support the findings of the case report have been provided in the article, which are available from the corresponding author upon reasonable request.

## Abbreviations

CRP: C-reactive protein
CT: Computerized tomography
IE: Infective endocarditis
MALDI-TOF: Matrix-assisted laser desorption ionization-time of flight
mNGS: Metagenomic next-generation sequencing
NT-BNP: N-terminal B-type natriuretic peptide
PCR: polymerase chain reaction
PCT: Procalcitonin
